# Research as a pillar of Lassa fever emergency response: lessons from Nigeria

**DOI:** 10.11604/pamj.2020.37.179.26425

**Published:** 2020-10-27

**Authors:** Adebola Tolulope Olayinka, Chioma Dan Nwafor, Adejoke Akano, Kamji Jan, Blessing Ebhodaghe, Kelly Elimian, Chinwe Ochu, Tochi Okwor, Oladipupo Ipadeola, Winifred Ukponu, Ifeanyi Okudo, Clement Peter, Elsie Ilori, Chikwe Ihekweazu

**Affiliations:** 1 World Health Organisation, Abuja, Nigeria,; 2 Nigeria Centre for Disease Control, Abuja, Nigeria,; 3 Georgetown University, Abuja, Nigeria

**Keywords:** Lassa fever, infectious disease outbreaks, operational research, health emergencies

## Abstract

Emerging and re-emerging infectious diseases are becoming more frequent and developing countries are especially at increased risk. A recurring infectious disease outbreak in Nigeria has been that of Lassa fever (LF), a disease that is endemic in Nigeria and other West African countries. Nigeria, between 1^st^ January and 27^th^ October 2019, reported 743 confirmed cases of LF and 157 deaths in confirmed cases. Lassa fever outbreaks continue to be recurrent after fifty years of its identification. The true burden of the disease in Nigeria is unknown while gaps in knowledge about the infection still persist. Based on the Nigeria national Lassa fever research agenda and the World Health Organisation's roadmap initiative for accelerating research and product development which enables effective and timely emergency response to LF disease epidemics among other infectious diseases; a research pillar was added to the seven existing LF emergency operations centre response pillars in 2019. We describe lessons learnt from the integration of a research pillar into the LF national emergency response.

## Commentary

Emerging and re-emerging infectious diseases are becoming more frequent and advancing faster than ever before, predominantly in developing countries of Africa, South America and South Asia [1]. Multiple factors have been associated with this trend such as biological, environmental, climatic and lifestyle changes, among others [2]. A recurring infectious disease outbreak in Nigeria has been that of Lassa fever (LF), a disease that is endemic in Nigeria and other West African countries including Sierra Leone, Liberia, Guinea and Benin. There is, however, increased risk of LF infection in neighbouring countries as the major animal vector for Lassa virus, *Mastomys natalensis* is distributed across this region [1].

In West Africa, LF is estimated to affect 100,000-500,000 persons annually; with Nigeria bearing the most burden [3] recording 177, 312 and 633 of laboratory confirmed cases from 2016 to 2018 [4]. In Nigeria, between 1^st^ January and 27^th^ October 2019, 743 confirmed cases of LF and 157 deaths in confirmed cases were reported [4,5]. Outbreaks of LF have been an annual occurrence in the country with increase in number of cases every subsequent year [5]. Series of preparedness and response activities of government organisations, public health agencies and health care centres to control the epidemic have not been very successful in preventing the recurrence of outbreaks of LF. Despite these, the true burden of the disease in Nigeria is unknown and many gaps in knowledge about LF still persist. The large LF outbreak in 2018 [5] was a pointer to this and at the 2019 Lassa Fever International Conference which held in January 2019 at Abuja, Nigeria, many questions were raised and the need to support further research on various aspects of Lassa fever was reiterated.

The World Health Organization (WHO) developed a roadmap initiative for accelerating research and product development to enable effective and timely emergency response to LF disease epidemics among other infectious diseases [6]. One of the key gaps identified was the need for effective coordination of research and development (R&D) to increase knowledge on LF and collectively facilitate the development of LF medical countermeasures (MCM). The scope of expected research ranges from basic research e.g. epidemiological, community perception, and evaluation studies to the randomized control trials of newly developed MCM to prevent and control Lassa fever outbreaks and endemic disease [6]. The recurring LF epidemic while highlighting the rising health system challenges in Nigeria and other affected communities has also exposed some intrinsic gaps in both knowledge and practice about the disease. The Nigeria Centre for Disease Control (NCDC) in 2018 with the support of the WHO developed a national Lassa fever research agenda; and therefore during the 2019 national response to the LF outbreak, a research pillar was added to the seven existing LF Emergency Operations Centre (EOC) response pillars. This write-up describes lessons learnt from the integration of a research pillar into the LF national emergency response.

**Overview of the national emergency response:** a cardinal point in the coordination of any emergency response to a public health event is the activation of an emergency operations or coordinating centre. In 2019, the national EOC for LF in Nigeria was activated on the 22^nd^ of January, following the declaration of the LF outbreak as an emergency by the NCDC due to high case numbers [7]. The EOC was set up with the response pillars of coordination, surveillance and epidemiology, case management, infection prevention and control and safe burial, laboratory, risk communication and logistics pillars. However, for the first time two additional pillars of research and vector control, food safety and environmental sanitation were set up during the 2019 response. The terms of reference for the research pillar were to identify, facilitate and support the conduct of research that would improve knowledge about LF and inform appropriate, evidence based prevention, response and control activities of the disease.

**Activities of the research pillar:** the NCDC LF research pillar commenced its activities by identifying and mapping ongoing research by various stakeholders across the country to avoid duplication of efforts. A 3-day capacity building workshop on the development of research proposals was also held for pillar members of the Lassa EOC. The research pillar participated in all meetings of the LF national EOC while also working closely with all the other response pillars to identify gaps from their operations which could be addressed by research. Reviewing daily and weekly reports from these other pillars of the EOC, gaps in knowledge, methods, response activities, assessments and challenges were identified as potential questions that could be addressed by research. Before the outbreak, WHO had supported the NCDC to prioritise its research areas with respect to LF and developed a research agenda. This was also used as a benchmark in the identification of potential research areas. During the outbreak, however, potential operational research that could immediately help to improve response were identified. These potential research areas spanned across case management, infection prevention and control, surveillance, contact tracing, laboratory, risk communication, environmental and vector control activities. The research pillar worked with the other response pillars concerned to develop concept notes around these gaps which would help inform their response subsequently. Those concept notes were prioritized based on the perceived impact on future prevention and response effects and a few of these concept notes were developed into research proposals for implementation by the rapid response teams (RRT) deployed to the field.

**Lessons learnt:** outbreak response activities help to highlight gaps that can be addressed by research which otherwise may be either overlooked or not have been noticed. An example is the actual infection prevention and control practice of healthcare workers during outbreak periods as opposed to during routine care at other seasons. Also having a research pillar helped provide needed guidance on response research activities at national and subnational levels leading to streamlined research activities thereby avoiding duplication of efforts and optimising the available resources. An example was in the utilisation of the same checklist and methodology for household assessment by the RRT across various states and local government areas they were deployed to. Another lesson learnt is the need to have supportive teams with enough research methodology capacity to rapidly develop scientifically sound proposals based on identified gaps during an epidemic response. This was a challenge for the team as there were limited members in the research pillar and the other response pillars were more focused on outbreak response activities rather than on development of proposals. Also studies will need to be conducted in such a way as not to disrupt the much needed response being provided by the RRT and other members of the various response pillars. For implementation, the RRT were saddled with implementation of the operational research in addition to response activities and this led to non-completion of data collection by many due to distraction by response activities. We also learnt that there is need to have pre-dedicated funding such as a certain percentage of outbreak operational funds to support research prior to predictable outbreaks. Some of the studies could not be implemented due to paucity of funds for research during the period and this limitation was also reason for the use of RRT to collect data; obtaining the support of ethics committees for expedited approval is crucial to the implementation of research during outbreaks as it has been noted that epidemics tend to reveal vital knowledge gaps that could neither have been predicted nor prioritized for study before an outbreak occurs [8]. Prior to this outbreak, WHO supported a meeting in conjunction with NCDC with both national ethics and regulatory committees on expedited review of protocols during infectious disease outbreaks. The response this year was better than previous but the advocacy needs to continue; there is considerable challenge also in having study teams or people who can move out to conduct the study while an outbreak is ongoing especially a viral haemorrhagic fever outbreak like LF; as most members of the response team are involved in the actual response efforts in multiple small teams which are already overwhelmed by response activities. We learnt these lessons from the research response pillar during the Lassa fever outbreak EOC in 2019.

**Recommendations:** gaps identified during an outbreak should continue to generate research questions for high-priority study as both an integral component of the immediate response and if not possible immediately, could be for longer-term follow-up study. This hopefully would help in both understanding of the disease process and inform best practice for prevention and response activities [9]; country preparedness activities should include research preparedness. Preparedness is an ongoing continuous activity and so this should happen with research also. Conducting research during an outbreak can be challenging as usually the focus is on responding to and ending the epidemic [10]. However, if enough preparation including allocated funding have been put in place before an outbreak commences, it would make it more feasible to conduct such research; in outbreak response, a research team with individuals that have capacity to rapidly develop sound operational research proposals across all pillars need to be identified and brought on board; operational research during outbreak creates many opportunities for the capture of data that could inform immediate response activities, but also benefit future preparedness and response efforts [9]; the period between epidemics needs to be dedicated to brainstorming, identifying research questions, proposal development, laying down the structure, getting ethical approval, identifying capacity and sourcing for funds for studies that would be conducted during the outbreak period; an integrated approach in which scientific stakeholders across disciplines come together during the pre-outbreak period to brainstorm and plan would be beneficial; implementation of social science and environmental research to further understand the particular behaviours that perpetuate Lassa virus transmission during both high and low transmission seasons is very important and would need dedicated researchers willing to stay within the communities for a long period of time to carry this out effectively and provide the much needed answers to this gap in knowledge; one health research teams need to be identified and networks formed where necessary in the inter-outbreak period as it takes time to build research teams especially when there is a need to build research capacity at the same time, this would help hasten the research process [10]; continuous capacity and capability building of response staff on implementation research is also recommended as this can help generate evidence to guide immediate and long-term interventions.

## Conclusion

Lessons learnt from the incorporation of a research pillar in the 2019 LF disease outbreak response provided an opportunity for both improvement and planning for research. This would inform future approaches to research-related response activities especially for predictable diseases like LF. This will ensure that we learn as much as we can from these significant public health events. The importance of utilizing research during an outbreak to evaluate and or improve our response and institute preventive programmes cannot be over emphasised; and this would go a long way in addressing knowledge gaps to improve prevention and control of outbreaks of infectious diseases such as LF.
